# Innovative Spine Implants for Improved Augmentation and Stability in Neoplastic Vertebral Compression Fracture

**DOI:** 10.3390/medicina55080426

**Published:** 2019-07-31

**Authors:** Francois H. Cornelis, Quentin Joly, Maud Nouri-Neuville, Mohamed Ben-Ammar, Bruno Kastler, Adrian Kastler, Nicolas Amoretti, Olivier Hauger

**Affiliations:** 1 Sorbonne Université, Department of Radiology, Tenon Hospital, 4 rue de la Chine, 75020 Paris, France; 2 Hyprevention, Clinical Research, 33604 Pessac, France; 3 Department of Adult Radiology, Necker University Hospital, 75015 Paris, France; 4 Neuroradiology and MRI unit, Grenoble University Hospital, 38700 La Tronche, France; 5 University of Grenoble Alpes, Grenoble Institute des Neurosciences, Inserm, U1216 Grenoble, France; 6 Department of Radiology, Centre Hospitalier Universitaire de Nice, Hôpital Pasteur 2, 30 Voie Romaine, 06000 Nice, France; 7 Department of Radiology, Centre Hospitalier Universitaire de Bordeaux, 33000 Bordeaux, France

**Keywords:** vertebral compression fracture, cancer, interventional oncology, bone, vertebroplasty

## Abstract

*Background and objectives:* Tumor-related vertebral compression fractures often result in severe back pain as well as progressive neurologic impairment and additional morbidities. The fixation of these fractures is essential to obtain good pain relief and to improve the patients’ quality of life. Thus far, several spine implants have been developed and studied. The aims of this review were to describe the implants and the techniques proposed to treat cancer-related vertebral compression fractures and to compile their safety and efficacy results. *Materials and Methods:* A systematic MEDLINE/PubMed literature search was performed, time period included articles published between January 2000 and March 2019. Original articles were selected based on their clinical relevance. *Results:* Four studies of interest and other cited references were analyzed. These studies reported significant pain and function improvement as well as kyphotic angle and vertebral height restoration and maintain for every implant and technique investigated. *Conclusions:* Although good clinical performance is reported on these devices, the small numbers of studies and patients investigated draw the need for further larger evaluation before drawing a definitive treatment decision tree to guide physicians managing patients presenting with neoplastic vertebral compression fracture.

## 1. Introduction

There are currently 1.4 million vertebral compression fractures (VCF) occurring over the world each year [1]. Origins of these fractures are diverse: Osteoporosis, trauma (burst fractures and other types of complex fractures), Kummel’s disease with associated osteonecrosis, as well as benign tumors (i.e. vertebral haemangioma) or cancer-induced lytic conditions such as malignant primary cancer (bone cancer, myeloma) or metastases [2,3,4]. Spine is the most frequent metastatic site, accounting for 39% of bone metastases [5]. Moreover, 5–10% of all cancer patients will develop metastatic spine tumors [6]. Spinal metastatic tumors are found in two thirds of cancer patients after cancer-related death [7]. 

As therapy improves the survival of cancer patients, more patients are thus in need for local treatments. Tumor-related VCF often result in severe and debilitating back pain as well as progressive neurologic impairment and additional morbidities including kyphosis, mobility impairment, and respiratory failure, worsening the functional and vital status of such fragile patients [8,9]. The fixation of these impending or actual fractures is thus essential to obtain good pain relief and subsequently to improve the patients’ quality of life. 

The treatment of spine metastases is often complex but aims at relieving pain, improving function, motricity, and patient’s quality of life, as well as improving survival. Management of spine metastases is based on a dual approach of systemic and local treatment strategies. Oncologic treatments of these tumors include radiotherapy, surgery, and palliative care [10,11,12]. For patients unfit for surgery, radiotherapy is often performed but doesn’t stabilize VCF [13,14]. Percutaneous vertebroplasty (PVP) is now considered as an effective option to stabilize the fracture and provide good pain relief in addition or not with radiation therapy [15]. As PVP presents up to 64% rate of cement leakage and up to 10% complications [16], balloon kyphoplasty (BKP) was developed to reduce the risks of cement leakage and to restore vertebral body height, while providing the same pain and function improvements as PVP. However, up to 38% of cement leakage has been reported, as well as incomplete vertebral body (VB) height restoration or loss of height after restoration, and possible adjacent fractures after BKP [17]. 

To provide better pain relief, function, and quality of life improvement, as well as long-lasting vertebral body height restoration, and to further limit the risks of cement leakage, several spine implants have been developed. The aims of this review were to describe the implants currently available to treat cancer-related VCF, their techniques, and to compile their safety and efficacy results available to date in the literature.

## 2. Materials and Methods

A systematic MEDLINE/PubMed literature search was performed with different combinations of terms as ‘‘vertebral compression fracture’’, ‘‘VCF’’, ‘‘KIVA’’, ‘‘Shield’’, ‘‘cement directed kyphoplasty”, “Osseofix”, “Vertebral Body Stenting” or “SpineJack”. Time period included articles published between January 2000 and March 2019. Original articles were selected based on their clinical relevance. Cited references from selected articles were analyzed to find and include significant papers previously excluded from our search or that did not come to our attention.

## 3. Results 

### 3.1. Vertebral Body Stent^®^

**Figure 1:**
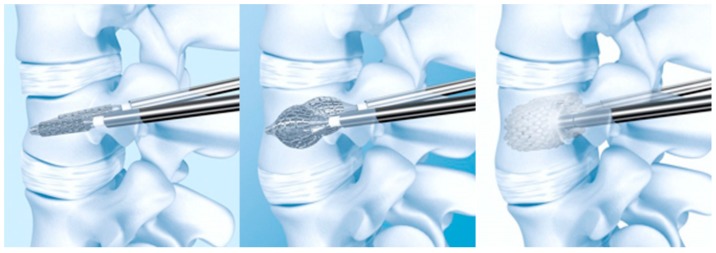
Vertebral Body Stent^®^ (VBS^®^) deployment procedure.

Vertebral Body Stent^®^ (VBS^®^) is a tubular-shaped metallic stent placed around an inflatable balloon tamp and designed to maintain the vertebral height restoration obtained after inflation of the tamp (Figure 1). The technique is pretty similar to BKP as two balloon tamps are introduced through a percutaneous transpedicular approach. As the balloon stamps are inflated, the metallic stents expand. After inflation, the metallic stents allow to maintain the cavity created by the balloon tamps and thus to maintain vertebral restoration while bone cement is injected [18]. 

Cianfoni et al. conducted a retrospective evaluation on 29 patients with 41 VCF with extreme osteolysis due to solid metastases, multiple myeloma or plasmacytoma, which aimed at evaluating the VBS^®^ efficacy with regard to spine stabilization [19]. All patients were implanted with VBS^®^ under biplanar fluoroscopic guidance, however some patients received additional posterior stabilization (1/29) or posterior stabilization and laminectomy (2/29). The results showed excellent VB height restoration in 75.6% of treated VCF, and 90% of VB height maintenance on a mean 15.3 months follow-up period (Table 1). No mention was made about pain and function in this study, but previous results on osteoporotic VCF showed a decrease of 4 to 6.4 Visual Analog Scale (VAS) points in favour of a significant pain relief and around 40% Oswestry Disability Index (ODI) score function improvement after 12 months [18,20,21]. VBS^®^ is thus considered a safe and effective option to treat lytic conditions-related VCF and maintain VB height improvement over time. Although limited by the number of patients included and the off-label use of the devices, a recent study suggested that cementoplasty combined with only a vascular stent deployed within the vertebral body could allow effective bone stabilization resulting in pain relief [22].

### 3.2. Osseofix ^®^

The principle of the Osseofix^®^ implant is similar to the one of the vertebral body stent: It consists of a tubular stent-like titanium mesh which is inflated mechanically without using a balloon tamp, unlike VBS and BKP. Its goal however is the same as this of VBS: To provide long-lasting VB height restoration and limit cement leakage as well as pain relief and function improvement. 

In 2014, Ender et al., published a prospective study aiming at establishing the usefulness of Osseofix^®^ to stabilize VCF in 32 patients [23]. Among them, eight patients with tumorous VCF were implanted with Osseofix^®^ under general anaesthesia with uniplanar fluoroscopic guidance and were followed for 12 months (Table 1). Results after 12 months showed a significant pain relief (VAS = –6.5 points), significant function improvement (ODI score = –40.7%), and significant kyphotic angle long-lasting improvement without perioperative or postoperative complications linked to the device or the procedure in this subgroup of patients [23]. Other studies performed on osteoporotic VCF patients report comparable pain, function, and radiological results [23,24,25].

### 3.3. KIVA^®^ Vertebral Compression Fractures Treatment System

**Figure 2:**
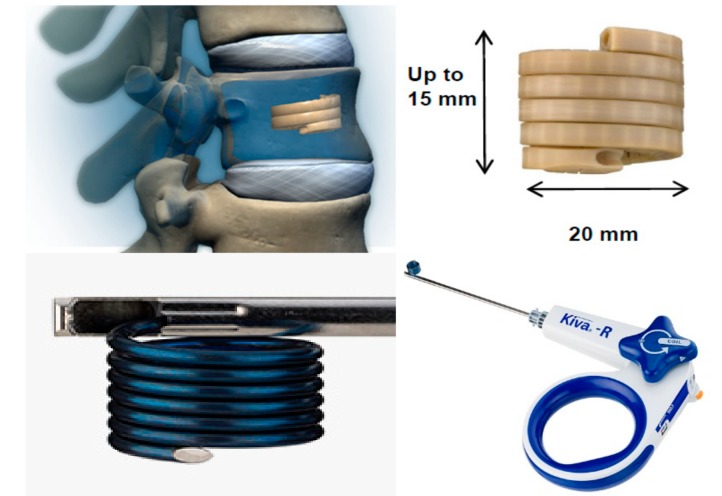
KIVA^®^ implant design and delivery ancillaries.

KIVA^®^ is an implant made of PEEK polymer (polyetheretherketone) which mimics the biomechanical properties of cortical bone. Its aim is comparable to the previous implants because its purposes are to mechanically reduce the fracture, to provide long-lasting VB height restoration and to avoid cement leakage [26]. In order to do so, KIVA^®^ is intended to be delivered percutaneously through a unipedicular approach over a removable nitinol coil in a continuous spiral loop into the vertebral body. A right and left pedicle version is available to provide the option to access the vertebral body from each pedicle. This design of delivery in a spiral loop shape allows to mechanically reduce the fracture: Each loop added to the spiral uplifts the vertebral endplate by a few millimeters, and the amount of the implant delivered is customized by the physician during the procedure (Figure 2). The tubular and perforated design of the implant allows injection of cement contained only in the inside of the spiral, thus preventing cement leakage. 

In 2013, Anselmetti et al. [27], presented a prospective study on 40 painful osteolytic spine malignancy patients with a 12-month follow-up after KIVA^®^ implantation which was performed under local anaesthesia and fluoroscopic associated to computerized tomography (CT) guidance. Results showed that on the long-term, the 40 patients experienced a mean nine points VAS score significant decrease, and a mean 78.1% ODI score significant drop. Radiological outcomes indicating VB height restoration or kyphotic angle modification was not assessed. Additionally, the study shows a 16.3% rate of cement leakage, and 3 new VCF due to additional spinal metastases (Table 1).

In a prospective comparative study published by Korovessis et al. [28], 23 patients with 41 VCF due to painful osteolytic vertebral metastases were treated using KIVA^®^, under general anaesthesia using biplanar fluoroscopy. Results showed a significant mean pain reduction of 5.1 points VAS score, and a mean significant ODI score improvement of 43%. However, no significant VB height restoration nor kyphotic angle improvement were reported at 1-month follow-up (Table 1). The main reason for the absence of significant restoration of kyphotic segmental vertebral deformity might reside in the fact that initial VB wedge deformity was moderate (around 13% in the KIVA^®^ group). No general nor surgical-related complications, as well as no cement leakage, were reported in this study for the KIVA^®^ group.

### 3.4. SpineJack^®^ Expansion Kit

**Figure 3:**
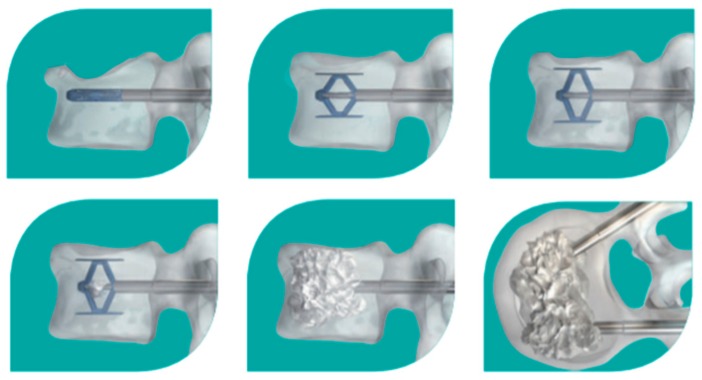
SpineJack^®^ implantation procedure

SpineJack^®^ implants are titanium devices based on the principle of a classic jack: When implanted into the vertebral body, the parasagittal-oriented SpineJacks allow, through activation of their mechanism, to apply a cradio-caudal moment in order to restore vertebral height, resulting in fracture reduction. Once the vertebral body height is restored, bone cement is injected to provide long-term fracture fixation (Figure 3). Although reported to provide significant pain reduction, ODI score improvement, vertebral body height restoration, vertebral body and Cobb’s angles restoration for trauma-induced and osteoporotic VCF in several studies [29], no studies were found investigating SpineJack as a treatment for neoplastic VCF.

### 3.5. V-STRUT^©^ Transpedicular Vertebral System

**Figure 4:**
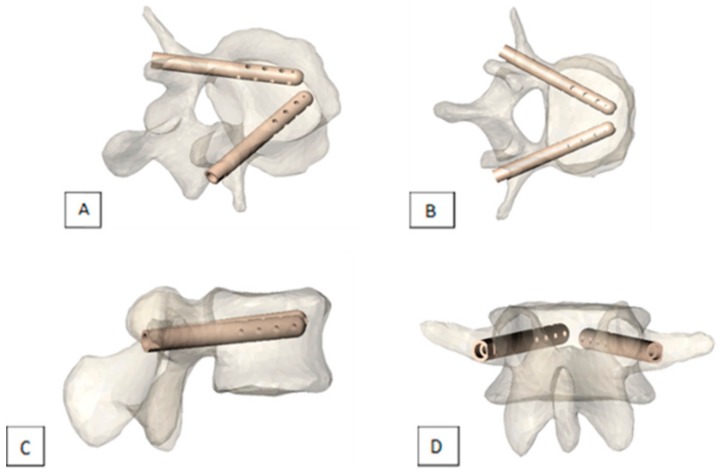
Views of V-STRUT^©^ implants in a vertebra (**A**) perspective, (**B**) top view, (**C**) side view, (**D**) back view

V-STRUT^©^ is a new implantable device designed for treatment or prophylactic fixation of VCF due to osteoporosis or osteolytic malignant bone lesions in the thoracic and lumbar spine (from T9 to L5 levels). It is made of radiotransparent PEEK polymer, with a cannulated and perforated design. Two devices per vertebra are implanted through a transpedicular approach. This device aims, as its competitors, at providing pain relief and function improvement as well as reducing cement leakage risks and maintaining VB height. Its cannulated and perforated design allows controlled cement injection in the vertebral body, thus reducing cement leakage risks while providing pain relief and function improvement by filling the lytic lesion. However, fracture reduction is not performed by the device itself, but rather performed if needed according to the practitioner by placing the patient in prone position. Its innovative aspect resides in the fact that its cannulated design and its posterior pedicle anchorage bring support to the superior vertebral endplate and allow to resist axial compression, thus avoiding reoccurrence of fracture [30] (Figure 4). 

**Figure 5:**
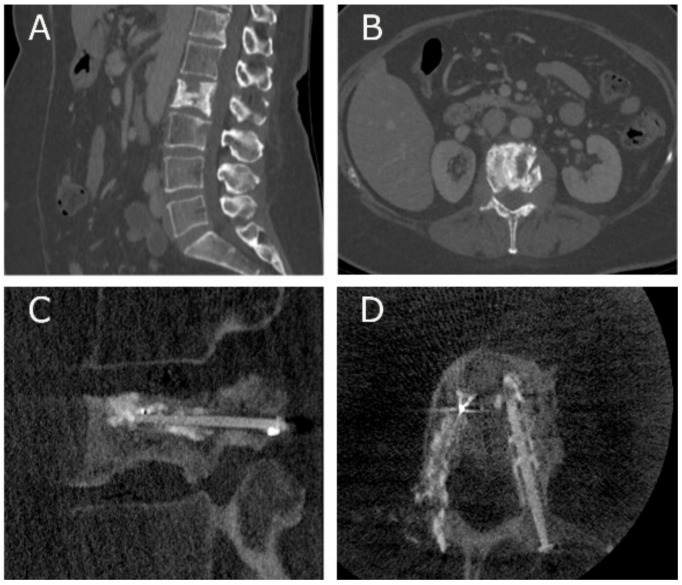
Pre-operative CT scans of the L2 metastatic lesion (**A**) sagittal, (**B**) transversal, and immediate post-operative fluoroscopic images of the PEEK (polyetheretherketone) implants (**C**) sagittal, (**D**) transversal.

An ongoing pilot multicentric study investigating the safety and efficacy of V-STRUT^©^, conducted by Cornelis et al. (ClinicalTrials ID: NCT03580434), enrolled the first patient in February 2019: A 59-year-old woman presenting a spinal metastasis at L2 level was treated using V-STRUT^©^. Preoperative CT scans showed partial vertebral collapse (Figure 5), associated with severe pain and mild function impairment (VAS = 7.0, ODI = 46%). The practitioner reported a 45 min implantation procedure, without technical difficulty, with approximately 5 cc of cement injected. Immediate postoperative fluoroscopy showed good implantation of the device (Figure 5), with the patient self-reporting absence of pain (VAS = 0) after the procedure. At the 2-month follow-up visit, the CT scan showed again good implantation of the device, and the patient still reported absence of pain.

## 4. Discussion

A small number of implantable devices are currently available on the market to treat neoplastic impending or pathological VCF. Despite the variety of designs and materials within this group of devices, studies demonstrated that they could provide significant pain relief, function, and motricity improvement similar to PVP and BKP [16,17]. These studies also highlighted the additional benefits provided by the investigated devices, such as reduction of cement leakage rates and maintainof vertebral body height restoration. Indeed, VBS demonstrated in 29 patients for 41 VCF 75.6% of estimated excellent reconstruction of vertebral height with a 97% estimated spine stabilization on a mean 15.3-month follow-up. VBS also demonstrated a lower rate of 34% of cement leakage compared with PVP and 4 asymptomatic adjacent fractures. Osseofix^®^ provided significant improvement in kyphotic angle on a 12-month follow-up within 8 patients, although the small number of patients may account for the absence of peri- or post-operative complications. KIVA^®^ implanted in 40 patients followed for 12 months was reported with a low rate of 16.3% of cement leakages and only 3 new spine fractures. However, on a small study of 23 patients followed for 1 month, KIVA^®^ demonstrated no significant VB height restoration and kyphotic angle improvement. This absence of deformity improvement was justified as the majority of VCF treated were impending VCF, with little to no baseline deformity. The KIVA^®^ device was just used in this study as a prophylactic VCF fixation solution. Ultimately, V-STRUT^©^ device is currently under clinical investigation but has demonstrated the feasibility of its procedure as well as good pain and function improvement in one patient followed for 2 months. All these preliminary results put together indicate that VBS, Osseofix^®^, KIVA^®^, and V-STRUT^©^ provide pain and function improvement, spine deformity restoration, and long-lasting maintain and limit adverse events occurrence, thus enhancing quality of life of cancer patients. Despite having been investigated in this context, SpineJack^®^ is indicated in the treatment of neoplastic VCF and has been reported to provide good clinical performance treating osteoporotic VCF.

Although presenting promising results, these studies limitations reside essentially in the small number of patients included and in their non-comparative (except for one study [28]) and non-randomized designs. The results presented in these studies must be considered with enthusiasm but also with care and circumspection. This systematic review of the literature presented only 4 relevant studies investigating implants used in the treatment of neoplastic VCF. To allow more reliable conclusions about the clinical performance of these devices, and to provide treatment guidelines and recommendations for treatment of pathologic VCF patients, further larger, randomized, and blinded comparative studies must be undertaken. 

## 5. Conclusions

Preliminary results from several studies demonstrated the feasibility, the efficacy, and the safety of using these above-mentioned devices for fixation or prophylactic treatment of pathological VCF. Although limitation to these preliminary results is the small number of patients enrolled in the studies, these devices appear as a promising alternative in fragile cancer patients. Further larger evaluation is mandatory before drawing a definitive treatment decision tree to guide physicians managing patients presenting with neoplastic vertebral compression fracture.

## Figures and Tables

**Table 1:** Results of published studies on implants used in vertebral augmentation procedures to treat pathological VCF.

Study	No. of Patients	Technique	Anesthesia	Guidance	Follow-up	Pain	Function	VB height	Complications
[19]	29 patients for 41 VCF	VBS	17 CS 12 GA	Biplane fluoroscopy	Mean 15.3m	NA	NA	75.6% excellent reconstruction 97% spine stabilization on follow-up	34% cement leakage 4 adjacent fractures, no symptoms
[23]	8 tumor patients	Osseofix	GA	Uniplanar fluoroscopy	12m	−6.5 pts	−40.7%	Significant improvement in kyphotic angle	No perioperative or postoperative complications in tumor patients
[27]	40 spine malignancy patients	KIVA	LA	Fluoroscopy/CT	12m	−9 pts	−78.1%	NA	3 new VCF due to additional metastases 16.3% cement leakage
[28]	23 patients for 41 VCF	KIVA	GA	Biplane fluoroscopy	1m	−5.1 pts	−43%	No significant improvement in VB height and kyphotic angle, due to little deformity preoperatively	No cement leakage No general or surgical complications

VB: Vertebral body, VCF: Vertebral compression fracture, CT: Computerized tomography
